# Accuracy of magnetic resonance imaging to detect cartilage loss in severe osteoarthritis of the first carpometacarpal joint: comparison with histological evaluation

**DOI:** 10.1186/s13075-017-1262-8

**Published:** 2017-03-14

**Authors:** Michael S. Saltzherr, J. Henk Coert, Ruud W. Selles, Johan W. van Neck, Jean-Bart Jaquet, Gerjo J. V. M. van Osch, Edwin H. G. Oei, Jolanda J. Luime, Galied S. R. Muradin

**Affiliations:** 1 Department of Radiology, Erasmus MC, University Medical Center Rotterdam, Rotterdam, The Netherlands; 2 Department of Plastic, Reconstructive and Hand Surgery, Erasmus MC, University Medical Center Rotterdam, Rotterdam, The Netherlands; 3 Department of Rheumatology, Erasmus MC, University Medical Center Rotterdam, Rotterdam, The Netherlands; 4 Department of Plastic, Reconstructive and Hand Surgery, University Medical Center Utrecht, Utrecht, The Netherlands; 5 Department of Rehabilitation Medicine, Erasmus MC, University Medical Center Rotterdam, Rotterdam, The Netherlands; 6 Department of Plastic, Reconstructive and Hand surgery, Maasstad Hospital, Rotterdam, The Netherlands; 7 Department of Orthopaedics and Department of Otorhinolaryngology, Erasmus MC, University Medical Center Rotterdam, Rotterdam, The Netherlands

**Keywords:** Magnetic resonance imaging, First carpometacarpal joint, Cartilage, Histology, Osteoarthritis

## Abstract

**Background:**

Magnetic resonance imaging (MRI) is increasingly used for research in hand osteoarthritis, but imaging the thin cartilage layers in the hand joints remains challenging. We therefore assessed the accuracy of MRI in detecting cartilage loss in patients with symptomatic osteoarthritis of the first carpometacarpal (CMC1) joint.

**Methods:**

Twelve patients scheduled for trapeziectomy to treat severe symptomatic osteoarthritis of the CMC1 joint underwent a preoperative high resolution 3D spoiled gradient (SPGR) MRI scan. Subsequently, the resected trapezium was evaluated histologically. The sections were scored for cartilage damage severity (Osteoarthritis Research Society International (OARSI) score), and extent of damage (percentage surface area). Each MRI scan was scored for the area of normal cartilage, partial cartilage loss and full cartilage loss. The percentages of the total surface area with any cartilage loss and full-thickness cartilage loss were calculated using MRI and histological evaluation.

**Results:**

MRI and histological evaluation both identified large areas of overall cartilage loss. The median (IQR) surface area of any cartilage loss on MRI was 98% (82–100%), and on histological assessment 96% (87–98%). However, MRI underestimated the extent of full-thickness cartilage loss. The median (IQR) surface area of full-thickness cartilage loss on MRI was 43% (22–70%), and on histological evaluation 79% (67–85%). The difference was caused by a thin layer of high signal on the articulating surface, which was interpreted as damaged cartilage on MRI but which was not identified on histological evaluation.

**Conclusions:**

Three-dimensional SPGR MRI of the CMC1 joint demonstrates overall cartilage damage, but underestimates full-thickness cartilage loss in patients with advanced osteoarthritis.

## Background

Osteoarthritis (OA) of the hand is the most prevalent disease of the hand joint, which can lead to pain and functional impairment. The disease is characterized by cartilage loss, subchondral bone changes and inflammation of the synovium. Despite the fact that changes in bone only are directly visible on conventional radiography (CR), and that joint damage on CR is only weakly associated with symptoms [1], it is the most widely used imaging method for assessing structural changes in hand OA in both clinical practice and clinical trials [2, 3].

Magnetic resonance imaging (MRI) is gaining popularity in studies of hand OA [4, 5] as it depicts bone, cartilage, and soft tissue changes, and images the complete joint in multiple planes. As a result, MRI has given us new insights into hand OA, such as the involvement of collateral ligaments [6, 7], the high prevalence of synovitis [8] and significant associations of joint pain with bone marrow lesions (BML) and synovitis [9, 10].

MRI of cartilage in hand OA has been less well-explored, yet accurate cartilage assessment would be a valuable addition to other pathological change detected by MRI in the assessment and follow up monitoring of the whole joint in hand OA. In studies of knee OA, quantification of cartilage using MRI is often an outcome measure in clinical trials, but cartilage imaging in the small joints of the hand is more challenging, as smaller voxel sizes are needed to depict the thin cartilage layer. Previous studies have reported that reliable quantitative evaluation of the cartilage layer in the small joints of the hand can be performed using conventional MRI and small dedicated coils [11, 12].

While in-vivo cartilage quantification with MRI in knee OA correlates well with histological findings [13, 14], to our knowledge, there are no reports in the literature of a comparison between in-vivo MRI cartilage assessment of the hand joints and histological evaluation. As surgery in hand OA is only regularly performed for treatment of OA at the base of the thumb, comparison between MRI and histological evaluation is only feasible in patients with symptomatic thumb base OA.

The aim of this study was therefore to quantitatively compare MRI-detected cartilage loss in patients with OA in the first carpometacarpal (CMC1) joint with histological evaluation.

## Methods

### Patients

We recruited 20 symptomatic patients who had been scheduled for trapeziectomy or hemitrapeziectomy to treat OA in the CMC1 joint. From April 2010 until October 2011 consecutive eligible patients at a University hospital and two teaching hospitals in the Netherlands were invited to participate in the study. The indication for surgery was based on severe pain and/or loss of function. Prior to surgery, patients underwent MRI and functional assessment of the thumb. Patients with previous surgery to the base of the thumb, or patients with contra-indications to undergoing MRI were excluded.

Patients were operated on by the surgeon treating them for hand OA. Additionally two healthy controls were included for comparison of MRI images only. This study was approved by the local ethics committees of the participating hospitals. All patients provided written informed consent prior to the investigation.

### MRI acquisition

MRI was performed using 3.0 T scanners (GE HD and GE Discovery MR750, GE healthcare, Milwaukee, Wisconsin). Patients were placed in the prone position with the arm extended above the head, the hand placed in the center of the magnet, and the thumb fully extended on a custom-made platform to stabilize and immobilize the hand. A custom-made 4.0-mm loop coil was placed on the dorsal side of the CMC1 joint and taped to the hand. Sagittal 3D fast spoiled gradient (SPGR) sequences with fat saturation (FS) were obtained with a spatial resolution of 0.1 by 0.2 mm (echo time (TE) minimal; field of view (FOV) 3–4 cm; frequency 256–320; phase 128–224; slice thickness 0.7 mm; bandwidth 15.6 kHz; two signals acquired). Proton density (PD)-weighted, fast recovery fast spin echo (FRFSE) sequences were acquired in the coronal and sagittal plane (repetition time (TR) 2400; TE 30; echo train length (ETL) 6; FOV 3–4 cm; frequency 256–320; phase 128–160; slice thickness 1.0 mm; bandwidth 15.6 kHz; three signals acquired). T2-weighted FRFSE sequences with fat saturation were obtained in the coronal direction (TR 3000; TE 68; ETL 6; FOV 4 cm; frequency 192; phase 128; slice thickness 2.0 mm; bandwidth 15.6 kHz; four signals acquired). The scanning acquisition time was 25 minutes.

### MRI evaluation

A series of evaluations were made of the MR images from patients in whom histological evaluation was not possible. In the first evaluation, we tested a scoring method for cartilage assessment similar to the MRI OA knee score (MOAKS) [15]. However, we decided not to use this scoring method as the cases tested all received the highest score possible, even though clear differences in cartilage damage were visible on the images. In the second evaluation we tested the currently used scoring method, which uses the same definitions as MOAKS for identification of partial-﻿t﻿hickness﻿ ﻿cartilage ﻿loss and full-thickness cartilage﻿ loss, but the extent of the cartilage damage is not scored on an ordinal scale from 0–3, but on a ratio scale from 0–100%. After the second evaluation we decided to score a thin layer of one or two voxels of high signal intensity (comparable to cartilage) on the bony surface area as partial-thickness loss, not full-thickness loss.

All images were evaluated by two musculoskeletal radiologists and a hand surgeon (GM, EO and HC) together in consensus. The readers were blinded to patient data, clinical data, histological findings and other imaging results. The anonymized images were read using the open source software ClearCanvas Workstation (ClearCanvas Inc., Toronto, Canada). Using all available sequences, the articular surface of the trapezium was evaluated for grade of cartilage loss as normal cartilage thickness, partial-thickness﻿ loss of cartilage, or full-thic﻿kness loss of cartilage. On each 0.7-mm SPGR FS slice the readers indicated the surface corresponding to each grade.

Measurements from all slices per patient were summed to compute the total articular surface, total area of normal thickness, total area of partial-﻿thickness loss, total area of full-th﻿ickness loss, and total area of any thickness loss (full and partial loss combined). Percentages of these were calculated for comparison with histological findings. The image quality of the SPGR images was scored as either low, sufficient for evaluation, or good. Low means that there is a reasonable chance that error was introduced because of the poor image quality.

The CMC1 joints were scored for presence or absence of osteophytes, erosions/cysts, and subluxation. Osteophytes were defined as abnormal bone formation in the peri-articular region on the SPGR and PD images. Erosions/cysts were considered as a single feature and were defined as sharply marginated bone lesions with increased signal intensity on SPGR images, and intermediate signal on PD images, which were visible in two planes. The joint was considered to be subluxated when 33% or more of the metacarpal surface area was not aligned with the trapezial surface area in the coronal or sagittal plane. Synovitis was not scored as we did not use a contrast agent.

### Tissue preparation

During surgery the trapezium bone was extracted as a whole or in multiple parts. If the trapezium was not extracted in one piece, care was taken that the articular area of the trapezium facing the first metacarpal bone was kept intact by splitting the trapezium horizontally leaving at least 5 mm of the distal trapezium intact. The resected trapezium was fixed in neutral buffered 10% formalin in the operating room. Trapezium bones were decalcified in formic acid. Large decalcified specimens were cut in half and all samples were embedded in paraffin. Each millimeter, a  5-μm-thick section was cut in the sagittal direction of the bone, mounted and stained with thionin [16].

### Histological evaluation

All histological sections were scored for cartilage damage by a trained researcher (MS). To determine the reproducibility of these scores, 10 patients were also scored by GvO, an experienced cartilage researcher. The scorers were blinded to the results of the MRI evaluation.

**Fig. 1:**
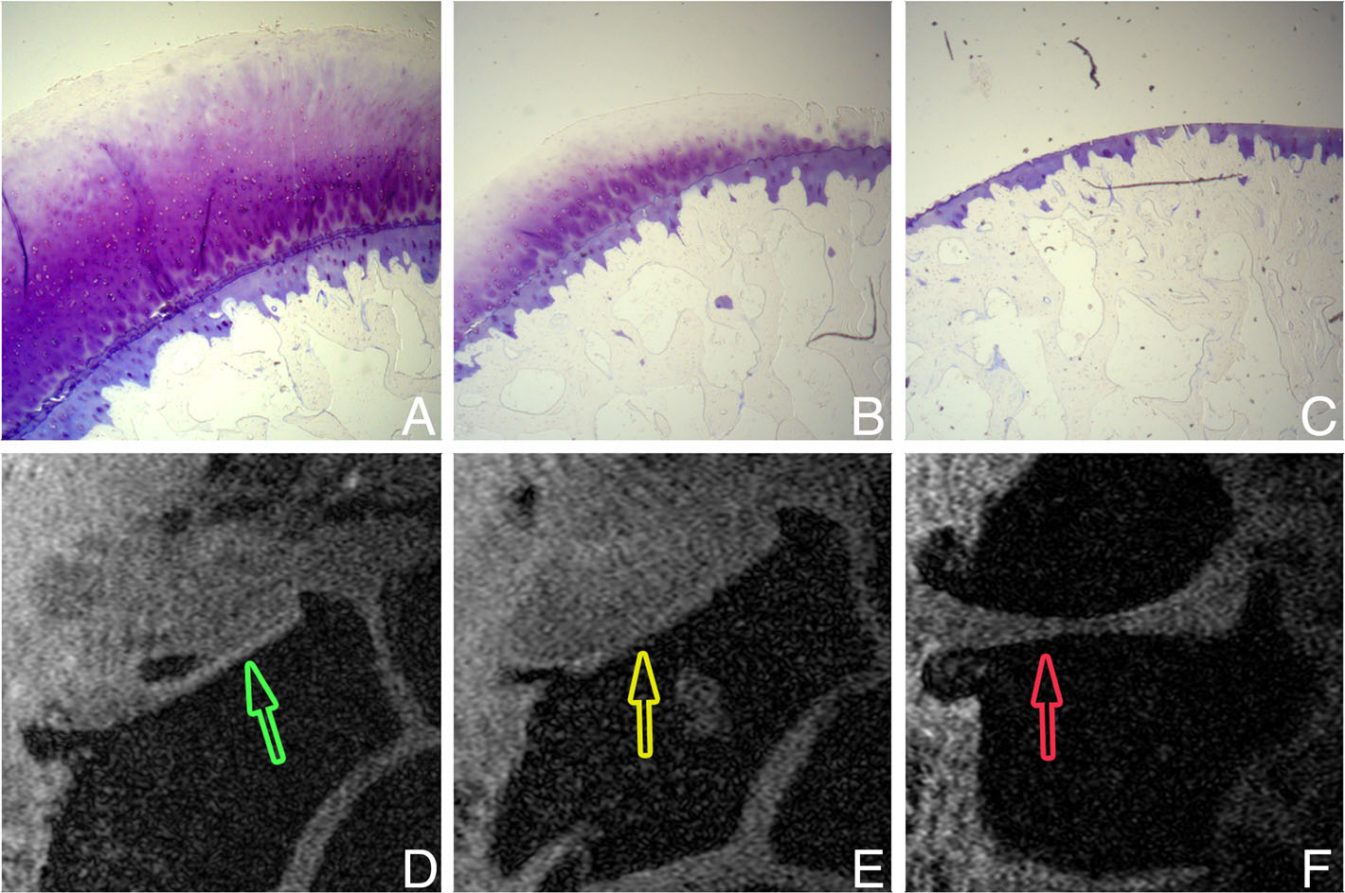
Example images of histological grading (**a**-**c**) and magnetic resonance imaging scoring (**d**-**f**), all from one patient. *Arrows* (**d**-**f**) indicate locations shown in **a**-**c. a**, **d** Cartilage of (near) normal thickness. **b**, **e** Partial-thick﻿ness loss of cartilage. **c**, **f** Full-thic﻿kness loss of cartilage. Due to subluxation in the joint, the metacarpal base is not seen in **d** and **e**. The quality of the magnetic resonance images was rated as good

All available sections were scored for severity and extent of cartilage damage. Severity of cartilage damage was scored according to the semi-quantitative grading and staging system devised by the Osteoarthritis Research Society International (OARSI) Working Group [17]. Grade, defined by depth of cartilage damage, and stage defined by the extent of horizontal cartilage damage were assessed. The OARSI grading system consists of six grades that describe increasing depth of damage to the cartilage damage. Grades 1–4 are subsequently described as: grade 1, edema or cell changes with an intact surface; grade 2, small surface discontinuities; grade 3, vertical fissures; and grade 4, delamination of the superficial zone. For comparison with MRI we defined grades 1–4 together as “cartilage with (near) normal thickness”. Grade 4.5 is described as mid-zone excavation, and was defined by us as “partial thickness loss of cartilage” for comparison with MRI. Grades 5 and 6 are described as: grade 5, complete erosion of hyaline cartilage to the level of mineralized bone; and grade 6, deformation and change in the contour of the articular surface. For comparison with MRI we defined grades 5 and 6 together as “full-thickness cartilage loss” (see Fig. 1 for examples).

Each histological section was scored for the amount of the articular surface that corresponded to each grade in decimals of percentage (i.e., either 0%, 10%, 20%, etc). The sum of the scores for each section had to be 100%. If there was no identifiable articular surface in a section, then no score was assigned to that section. Finally, all section scores per patient were averaged to calculate the total percentage area of (near) normal cartilage thickness, partial loss of cartilage thickness, and full loss of cartilage thickness.

### Statistical analysis

Descriptive statistics were used to describe the results of MRI and histological evaluation. Inter-reader reliability of the histology scores was calculated using the intraclass correlation coefficient (ICC). The ICC values were calculated as two-way random, single measures of absolute agreement [18].

## Results

### Patients

Twenty patients and two healthy controls were included in the study. In five patients, the trapezium was very deformed and could not be extracted without severely damaging the distal articular surface. We were therefore unable to obtain histological specimens from these patients. During histological analysis of the 15 specimens, we noticed that a considerable part of the articular surface was missing in the specimens of 3 patients. These patients were excluded from further analysis. The MRI scans of the excluded patients were used for training and calibration of the MRI score.

The final patient group consisted of 12 patients; two were male and 10 were female, with an average age of 60 (range 46–77) years. The median number of days between MRI and surgery was 8 (range 1–39). Mean grip strength (SD) was 23 (11) kg and mean pinch strength (SD) was 3.8 (0.9) kg. Self-reported pain assessed by visual analog score (possible range 0–100) varied widely between patients. The median (IQR) pain score at rest was 19 (5–31), and the median pain score during thumb movement was 57 (37–67).

### MRI

The image quality in 8 out of our 12 patients was adequate or higher, but was low in the other 4 patients. All patients had one or more osteophytes at the trapezium. All but one patient had cysts and/or erosions on the trapezium, and 7 out of 12 CMC1 joints were malaligned or subluxated. Overall cartilage damage was severe (Table 1). All patients had at least one small area with full-thickn﻿ess cartilage loss; 5 out of 12 patients did not have any remaining area of cartilage of normal thickness. The median (IQR) surface area of trapezial cartilage damage was 98% (82–100%). The percentage area with full-thickness loss of cartilage was 43% (22–70%). The image quality in both healthy controls was good, and they both had normal cartilage layers, without any damage.

**Table 1:** Histological and MRI scores for each individual patient

Patient	Histological evaluation			MRI			
	Normal	Partial-th﻿ickness loss	Full-th﻿ickness loss	Normal	Partial-thick﻿ness loss	Full-th﻿ickness loss	Image quality
1	0	0	100	28	45	27	Adequate
2	6	9	85	0	44	56	Low
3	0	25	75	0	77	23	Adequate
4	2	10	88	17	68	15	Adequate
5	22	30	48	37	54	9	Good
6	0	15	85	14	15	71	Adequate
7	4	17	79	2	31	68	Adequate
8	25	22	53	35	22	43	Good
9	10	31	59	19	61	20	Adequate
10	1	18	82	0	74	26	Low
11	3	11	86	0	26	74	Low
12	16	10	74	0	7	93	Low

For both histological evaluation and magnetic resonance imaging (MRI) the percentages of the articular surface that were normal, had partial-t﻿hickness﻿ loss of cartilage, or had full-thic﻿kness loss of cartilage, and MRI image quality are shown

### Histological evaluation

The mean number of histological sections acquired from each trapezium containing articular surface was 10 (range 9–14). Ten patients were scored independently by both readers. For inter-reader reliability of the detection of any cartilage loss over all scored sections containing articular surface (*n* = 100) was ICC = 0.70 (95%CI = 0.53-0.81), and the inter-reader reliability over all sections for full cartilage loss, the ICC was 0.84 (95% CI 0.76–0.90). Overall cartilage quality was poor (Table 1). No patient had any normal healthy cartilage remaining. The best cartilage observed had a histological grade of 3, with vertical fissures extending from the surface into the mid zone and depletion of matrix staining in the upper half of the cartilage. In 11 out of 12 patients there was complete erosion of the cartilage on more than half of the articulating surface. The median (IQR) surface area of trapezial cartilage damage was 96% (87–98%). The percentage area with full-thickness loss of cartilage was 79% (67–85%).

**Fig. 2:**
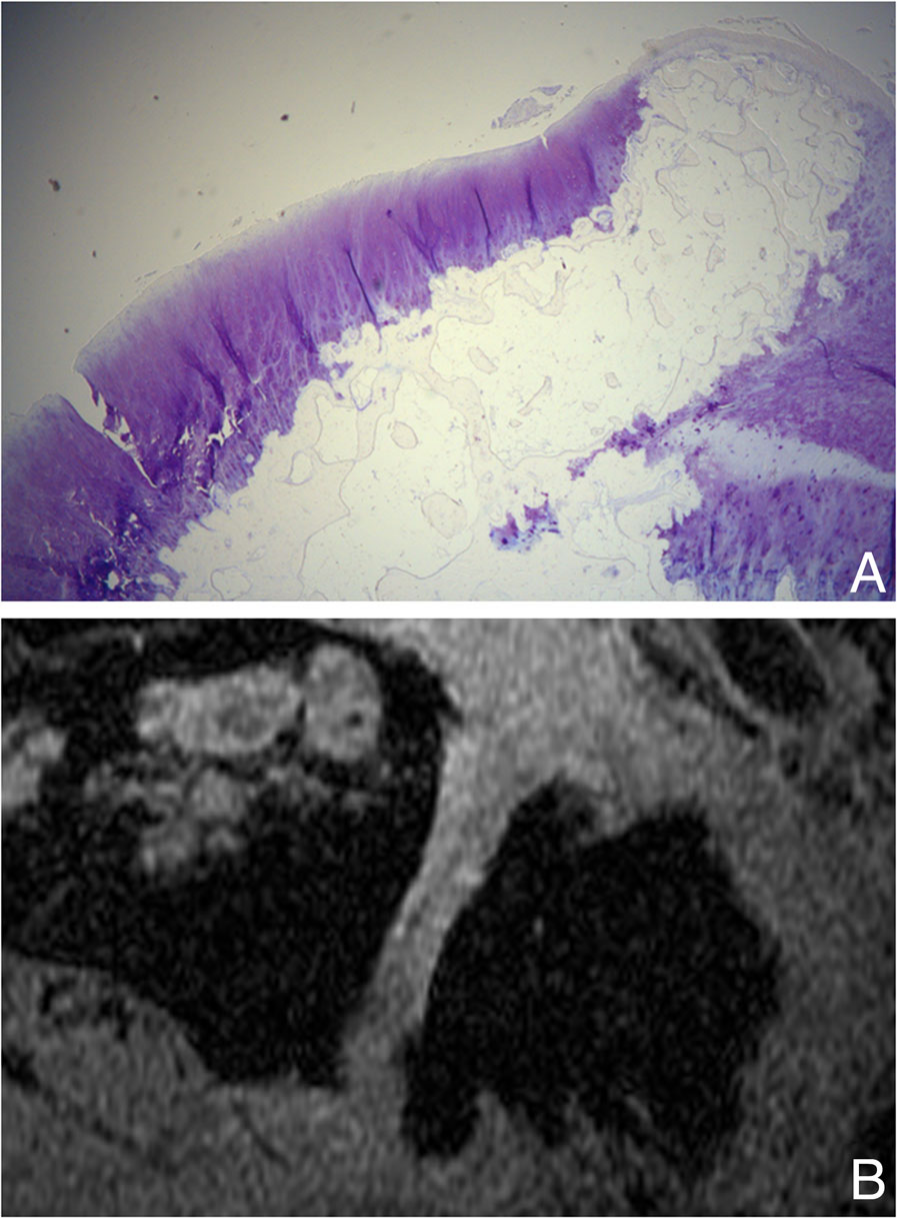
**a** Part of a histological section obtained from one of the patients. An osteophyte is visible on the right side. The remaining cartilage partially continues to cover the articulating surfaces of the osteophyte. **b** Spoiled gradient magnetic resonance image of the same patient, where the same osteophyte is on the upper side of the trapezium. Cartilage is visible in the center of the articulating surface of the trapezium and partially continues to cover the osteophyte, comparable with the histological image

After analysis, the largest differences between histological scores were in areas near osteophytes, which were sometimes partly covered with cartilage (Fig. 2). For scoring purposes osteophytes were excluded from the articular surface, and the cartilage formed on top of osteophytes was ignored. The lack of a clear anatomical landmark between the articular surface and osteophytes was the main cause of variations in scoring, as the region where the articular surface stopped and the osteophyte began was inconsistently scored.

### MRI vs histological evaluation

**Fig. 3:**
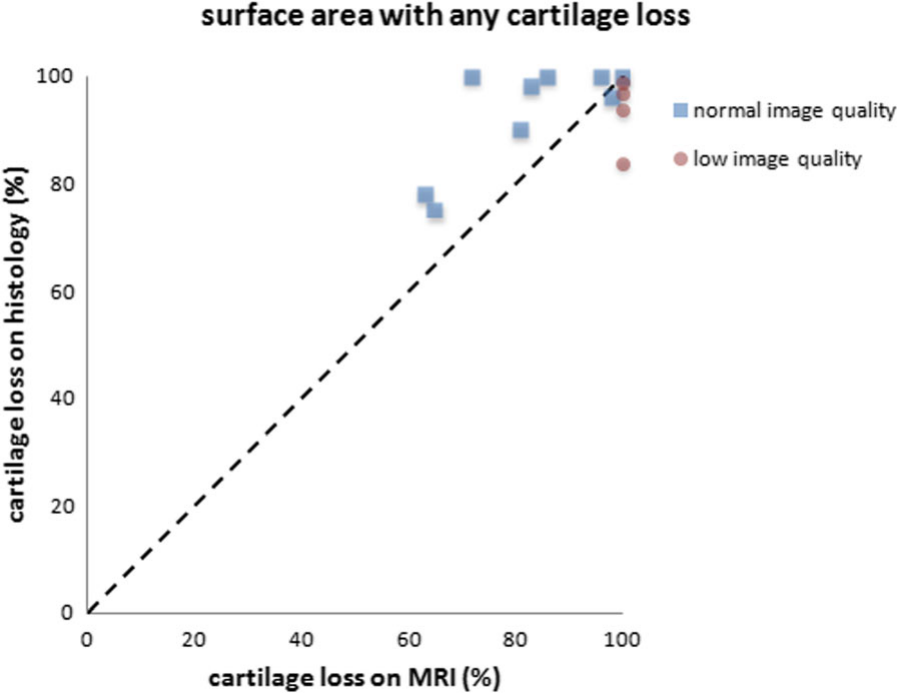
Relative area of the trapezial articular surface with any loss of cartilage identified. Each *dot* represents one patient measured by magnetic resonance imaging (*MRI*) and histological evaluation. Perfect agreement would result in all dots being positioned on the *diagonal line*

**Fig. 4:**
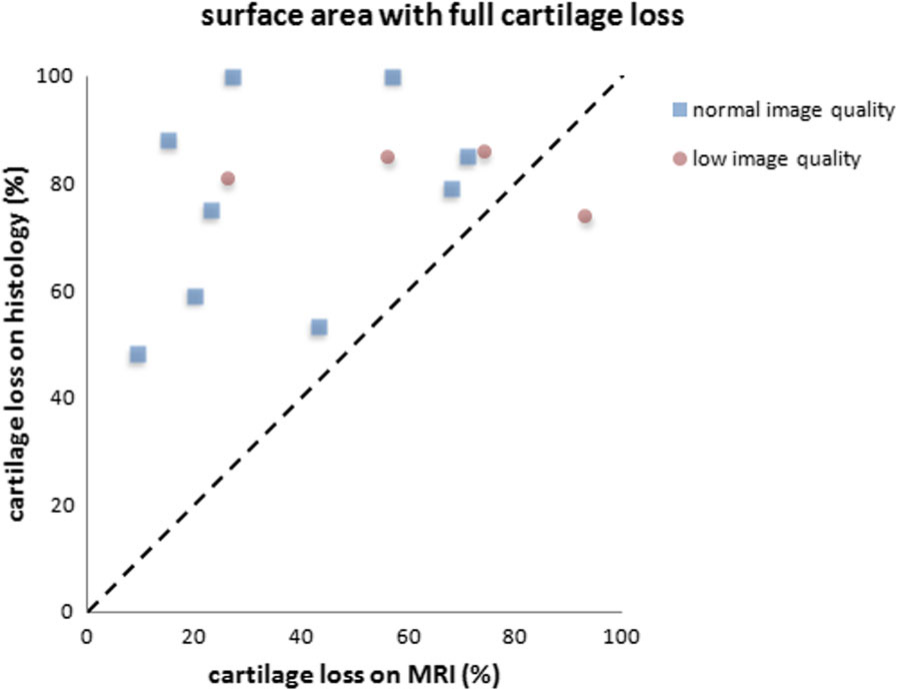
Relative area of the trapezial articular surface with full-thickness loss of cartilage identified. Each *dot* represents one patient measured by magnetic resonance imaging (*MRI*) and histological evaluation. Perfect agreement would result in all dots being positioned on the *diagonal line*

**Fig. 5:**
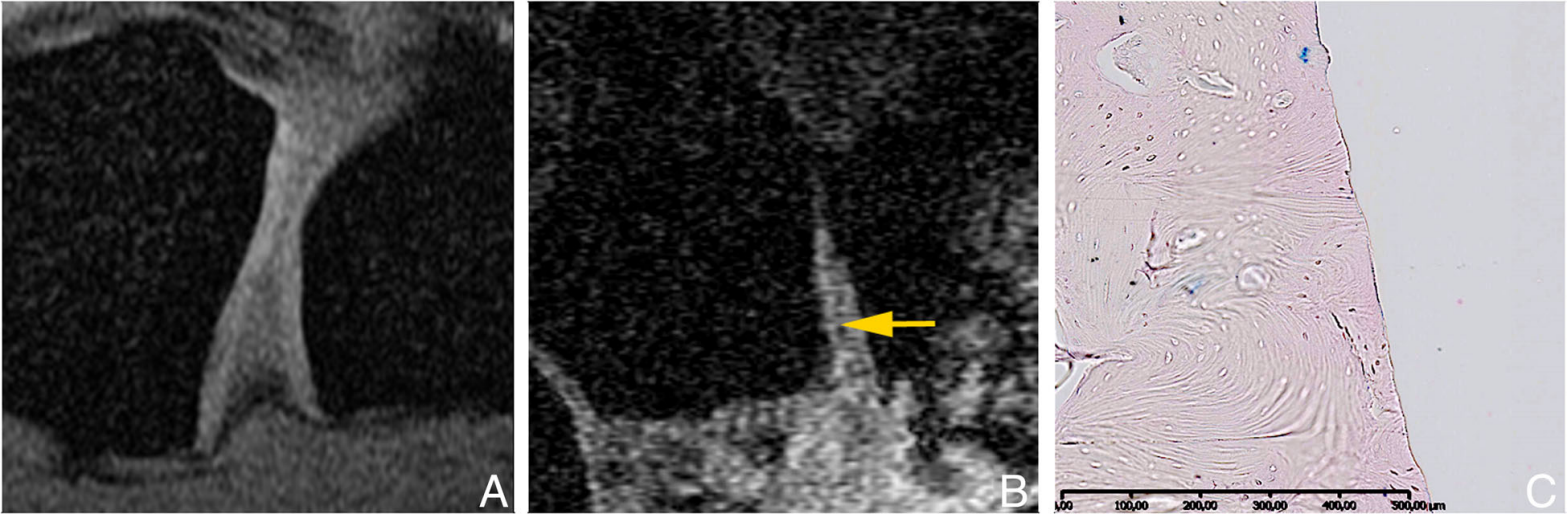
**a** Zoomed spoiled gradient, magnetic resonance image with fat saturation, of the first carpometacarpal joint (CMC1) in a healthy volunteer, showing a thick cartilage layer with high signal intensity. **b** Image of the CMC1 joint in a patient. *Arrow* indicates a thin band of high signal intensity, which was scored as partial loss of cartilage thickness (there appears to be some cartilage remaining). The image quality was rated as adequate. **c** Magnification of a histological section from the same patient; each *tick* on the *scale bar* represents 50 μm. The whole articular surface area in this patient had the same appearance, with only bare bone apparent

Both MRI and histological evaluation identified large areas of cartilage loss, with histological evaluation identifying slightly larger areas compared with MRI. The individual scores for each patient obtained by the two modalities are represented in Fig. 3. Histological evaluation identified substantially larger areas with full loss of cartilage than MRI (Fig. 4). Retrospective direct comparison of SPGR images and histological sections showed that the difference between MRI and histological evaluation in scoring any cartilage loss could in most cases be attributed to a thin layer of high signal intensity on the bony surface, which was scored as cartilage on MRI, but was not identified as cartilage on histological evaluation (Fig. 5).

Image quality for MRI was scored as low in 4 out of 12 patients due to motion artifacts and inability to place the surface coil in the optimal position because of disfigurement of the joint. However, we did not find a relationship between image quality and discrepancies between MRI and histological evaluation.

## Discussion

Our study showed that the overall extent of cartilage loss in small joints of the hand could be detected with 3D SPGR MRI. However, MRI underestimated the area of full-thickness loss of cartilage.

Previous studies have shown that the SPGR sequence is an accurate sequence to image cartilage in the knee joint [19, 20]. While it has been shown that SPGR may overestimate cartilage damage in early OA due to magnetic field inhomogeneity artifacts, considerable underestimation of cartilage damage has not been reported.

In previous studies assessing the accuracy of detection of cartilage defects and/or cartilage volume in the knee using MRI, the patient group either consisted of patients with relatively little damage, [19, 21–23] or the areas with severe cartilage damage were not analyzed [14, 24]. In the studies of patients with knee OA and relatively little cartilage damage, SPGR MRI had high sensitivity and specificity for detecting cartilage lesions in comparison with arthroscopy [19, 23] and very good correlation with cartilage thickness in comparison with histological evaluation [22].

The underestimation of full cartilage loss with MRI was caused by the thin layers of high signal on the articular surface that were visible on SPGR MRI, which were interpreted as thin layers of damaged cartilage. On retrospective comparison of the acquired SPGR and PD images and histological evaluation, the thin layers of high signal intensity on SPGR images were not identifiable on the PD images, and histological examination identified bare bone at the corresponding locations. These thin lines of high signal intensity adjacent to subchondral bone have previously received little attention in knee OA, as the line is very thin compared to the thicker knee cartilage, and has been counted as full loss of cartilage thickness in MRI knee OA studies [25]. The same kind of thin lines were previously described by Yoshioka et al. [26] in healthy volunteers on the posterior region of the femoral condyle within normal cartilage. The origin of this line is unclear. In our study it may have been caused by an artifact, but we cannot exclude the possibility that it represents a real anatomical substrate, such as a loose-lying layer of thin soft tissue, which may be lost during histological preparation.

We recognize that our study has limitations. First, the study design required patients to be scheduled for trapeziectomy, limiting the spectrum of disease severity. However, this is the only feasible method for acquiring in vivo histological specimens of cartilage from the small joints of the hand. To maximize the variation in cartilage status between our subjects, we included all patients undergoing trapeziectomy for treatment of pain and functional impairment, irrelevant of the severity of OA as determined radiographically. While we expected to also include some patients with mild cartilage damage, all our patients had severe cartilage damage on histological evaluation. Patients with milder OA or pre-clinical OA will have less damaged cartilage, but as mild thinning of the cartilage was also detectable in the less damaged areas of the joints in our patients, we expect that the imaging method can be used in patients with less severe OA.

The second limitation concerns image quality. Four out of twelve of our MRI examinations were of poor image quality, which may have impacted the MRI results of these four patients. Our coil was a loop coil with a diameter of 40 mm, which was optimal for imaging the CMC1 joint in healthy volunteers. However, in our patients with CMC1 OA, the distance between the coil and the center of the joint was larger because of the presence of osteophytes and subluxation, and the inability of patients to hold the thumb in full extension for optimal coil placement, reducing signal-to-noise ratio. Motion artIfacts also had a big impact on image quality. Improvements in either patient/coil positioning or the coil itself should be able to increase overall image quality.

The third limitation concerns the chosen MRI pulse sequence. We chose to assess cartilage with a 3D SPGR fat-suppressed pulse sequence for its high in-plane resolution with thin 0.7-mm slices, to be able to detect small cartilage lesions. This pulse sequence has previously shown promising results in the small joints of the finger [11, 27]. In healthy volunteers this sequence clearly delineated high-signal cartilage layers. In our study population of patients with advanced osteoarthritis only and with histologically proven abnormal cartilage, the signal intensity of cartilage was lower than expected based on MRI in healthy volunteers. Our MRI readers, therefore, sometimes had trouble delineating the cartilage from the joint fluid, which is a known disadvantage of this pulse sequence [28, 29]. While this will have introduced some error in the results, this was often resolved after crosschecking with the PD and T2 FSE sequences to make the distinction between fluid and cartilage. In this study we did not detect any small focal areas of cartilage loss, raising the question whether such thin slices are required to evaluate cartilage damage in advanced OA. Other pulse sequences such as dual-echo steady state (DESS), SPGR with iterative decomposition of water and fat with echo asymmetry and least-squares estimation (IDEAL), and true fast imaging with steady state precession (TrueFISP) have been found to have better cartilage-to-fluid contrast in the knee joints in healthy volunteers [28, 29]. If these sequences can be adequately optimized for the small FOV and high resolution, they may improve accuracy for detecting cartilage damage in the small joints of the hand.

Our MRI scoring method worked in a small number of patients, but is too time-consuming for larger studies. We chose this method to be as accurate as possible, but would not advise it for use in larger studies; instead, either automated segmentation for detailed detection of cartilage damage or a semi-quantitative score would probably be better.

## Conclusion

Three-dimensional SPGR MRI of the carpometacarpal joint of the thumb is able to detect the overall extent of cartilage damage. However, in severe cartilage damage, a layer of high signal intensity on the bone can be seen on 3D SPGR MRI, which does not always correspond to cartilage on histological evaluation, and could therefore lead to overestimation of the remaining cartilage.

## Abbreviations

BML: Bone marrow lesions
CMC1: First carpometacarpal joint
CR: Conventional radiology
ETL: Echo train length
FOV: Field of view
FRFSE: Fast recovery fast spin echo
FS: Fat suppression
ICC: Intraclass correlation coefficient
MOAKS: Magnetic resonance imaging osteoarthritis knee score
MRI: Magnetic resonance imaging
OA: Osteoarthritis
OARSI: Osteoarthritis Research Society International
PD: Proton density
SPGR: Spoiled gradient
TE: Echo time
TR: Repetition time
